# Evaluation of Lead Release in a Simulated Lead-Free Premise Plumbing System Using a Sequential Sampling Approach

**DOI:** 10.3390/ijerph13030266

**Published:** 2016-02-27

**Authors:** Ding-Quan Ng, Yi-Pin Lin

**Affiliations:** 1 Department of Civil and Environmental Engineering, Faculty of Engineering, National University of Singapore, Singapore 117576, Singapore; ngdingquan@gmail.com; 2 Graduate Institute of Environmental Engineering, National Taiwan University, No. 1, Sec. 4, Roosevelt Road, Taipei 10617, Taiwan

**Keywords:** copper, corrosion, brass, orthophosphate, sampling protocol

## Abstract

In this pilot study, a modified sampling protocol was evaluated for the detection of lead contamination and locating the source of lead release in a simulated premise plumbing system with one-, three- and seven-day stagnation for a total period of 475 days. Copper pipes, stainless steel taps and brass fittings were used to assemble the “lead-free” system. Sequential sampling using 100 mL was used to detect lead contamination while that using 50 mL was used to locate the lead source. Elevated lead levels, far exceeding the World Health Organization (WHO) guideline value of 10 µg·L^−1^, persisted for as long as five months in the system. “Lead-free” brass fittings were identified as the source of lead contamination. Physical disturbances, such as renovation works, could cause short-term spikes in lead release. Orthophosphate was able to suppress total lead levels below 10 µg·L^−1^, but caused “blue water” problems. When orthophosphate addition was ceased, total lead levels began to spike within one week, implying that a continuous supply of orthophosphate was required to control total lead levels. Occasional total lead spikes were observed in one-day stagnation samples throughout the course of the experiments.

## 1. Introduction

Lead is a toxic metal with a Maximum Contaminant Level Goal (MCLG) of zero mg/L; no amount of lead consumed is safe for human health. There is no reference dose (RfD) provided by the United States Environmental Protection Agency (USEPA) Integrated Risk Information System (IRIS) based on the same reason. Ingestion of lead can cause serious gastrointestinal and neurological problems, especially in children and fetuses [1,2,3]. To safeguard public health, the use of lead pipes in the distribution system has been banned since the 1980s in many countries. For example, lead pipes were banned in the US under the Safe Drinking Water Act in 1986 [4], followed by the implementation of the Lead and Copper Rule (LCR) in 1991, which set an action level for lead at 0.015 mg/L [5]. It is important to note that the action level is not a health-based standard, but a regulatory standard which triggers water utility actions when the 90th percentile exceeds the action level. The guideline value for lead in drinking water set by the World Health Organization (WHO) is 0.01 mg/L [6].

Although lead pipes are no longer installed in new systems, many older districts still contain lead service lines or leaded solders in their distribution systems [7,8,9,10,11]. Recent cases of elevated lead in tap water in cities without lead pipes have been attributed to galvanic corrosion of lead/copper coupling and metal alloys such as brass. In 2006, tap water in Durham, North Carolina, was found to contain more than 800 µg/L lead despite having no lead pipes in the system [12]. The source of lead was traced to a lead solder and the release of hazardous levels of lead was attributed to a change in coagulant from aluminum sulfate to ferric chloride in the water treatment plant. This change of the coagulant increased the chloride-to-sulfate mass ratio (CSMR) and enhanced galvanic corrosion of the lead solder [13]. Anion exchange for arsenic and organic anion removal, which releases chloride to the treated water, can also result in a high CSMR, increasing the corrosion potential for lead [14]. However, recent studies reported higher lead release at higher chloride and sulfate concentrations at a constant CSMR, indicating that the CSMR may not be a good indicator for lead corrosivity in water [14,15].

Galvanic corrosion also occurs during partial lead pipe replacements with copper pipes [7] and in brass materials, which are commonly found in premise plumbing systems [16,17]. In effect from 4 January 2014, the definition of “lead-free” by the USEPA has been revised to not more than a weighted average of 0.25% lead in wetted surface [18], but this regulation does not affect plumbing materials already in use, which followed the old “lead-free” definition that allows up to 8% lead by mass [4]. Lead is used in small amounts (<8% lead by mass) in brass manufacturing to increase machinability and make casting pressure tight by sealing the voids created during cooling [19]. Lead can also be present in brass as a contaminant from the recycling of materials [20]. Elfland *et al*. [17] showed that certified ball valves could contain a significantly higher lead mass ratio on the surface than in the whole valve. 

Under the LCR, the first-draw 1-L samples after a stagnation period of at least 6 h are collected by customers at the point of use [21]. This approach can detect lead release within the household, but it does not detect lead release in lead service lines (LSLs) [22]. In Europe, large numbers of random daytime (RDT) samples (without stagnation) and samples taken after a fixed stagnation time of 30 min were collected to provide a reasonable estimate of the average lead concentration at the tap [23,24]. However, RDT sampling is not reproducible and a large number of samples would be required to gauge the effectiveness of corrosion control [25]. In Canada and France, sampling is conducted by trained technicians with a stagnation time of 30 min [26,27].

In Singapore, there is no lead pipe or lead solder in the drinking water distribution system. Brass fittings are, however, commonly found in premise systems in many households. Brass fittings have been reported to be potential lead sources in works by Kimbrough [16] and Elfland *et al*. [17]. In this study, a simulated premise plumbing system was built using locally (Singapore) available plumbing materials to investigate lead release from “lead-free” plumbing materials. Particularly, the objectives are to identify any potential lead sources of the “lead-free” system using a modified sampling method and to evaluate the effectiveness of using orthophosphate for lead release control. The practicability and relevance of orthophosphate addition in premise plumbing system will also be discussed. 

## 2. Materials and Methods

### 2.1. Simulated Premise Plumbing System

**Figure 1:**
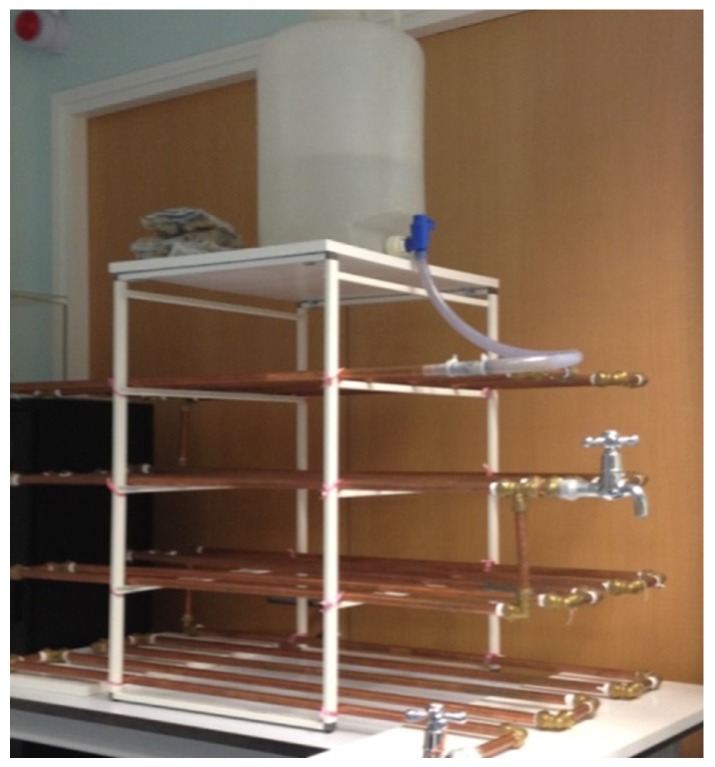
Photograph of the simulated lead-free premise plumbing system.

To determine whether lead contamination in drinking water will occur in a “lead-free” premise plumbing system, a typical premise system was built using locally (Singapore) purchased materials as shown in Figure 1. In Singapore, all water fittings used shall conform with the standards and requirements stipulated by the Public Utilities Board (PUB) and their use shall conform to the Public Utilities (Water Supply) Regulations and Singapore Standard Code of Practice (CP) 48. A water fitting shall be deemed to comply with the stipulated standards if it is certified by an accredited product certification body. All metallic material in contact with water shall comply with the test on extraction of metals of AS/NZS 4020:2005 in which leaching test was conducted for 24 h at 80 °C. The maximum allowable concentrations of metals leached shall not exceed the limits specified by the WHO Guidelines for Drinking Water Quality [28,29]. Copper pipes and brass fittings used in this study were microwave-digested and measured for lead content, and results showed that their lead content was <0.25% by weight, and thus can be considered “lead-free” according to the last “lead-free” definition by USEPA.

**Figure 2:**
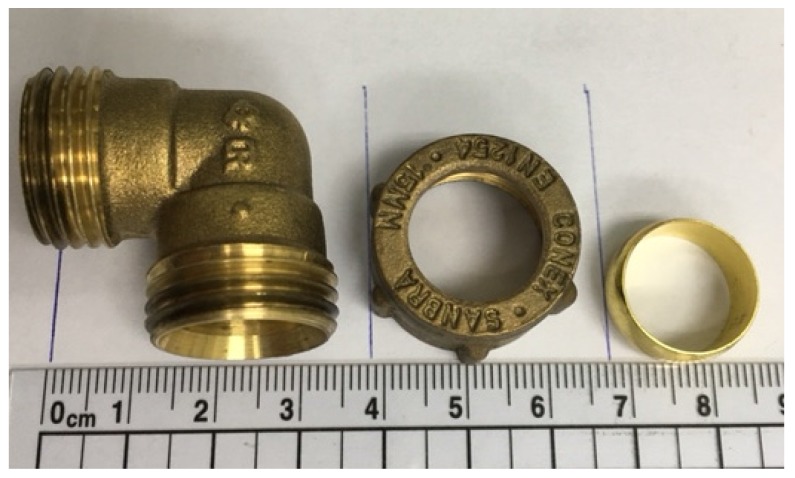
Photograph of brass fitting assembly: body (**left**); compression nut (middle) and compression ring (**right**).

The system consists of approximately 20 m of certified (BS EN1057:2006) copper pipes (0.5 inch dia), (BS EN1254-2:1998) brass fittings (Figure 2) and stainless steel taps. The required copper pipe length is based on the average household floor area of about 95 m^2^ in Singapore [30]. The total capacity of the system is approximately 2.8 L. The first stage of the experiment (conditioning phase) was to identify a potential lead source in the system using a modified sampling method. The second stage was to examine the effects of orthophosphate, a common corrosion inhibitor, on lead release from the identified lead source. The third stage was to assess the effects of stopping orthophosphate addition on lead release. 

Tap water (Table 1) collected from the laboratory was used. A 25 L plastic container was used to hold supply water for the system. Plastic tubing was used to connect the plastic container to the copper pipes and brass fittings were used to join the pipes and taps. Sequential sampling of first 200 mL using either 2 × 100 mL or 4 × 50 mL glass bottles were performed after one, three and seven days of stagnation. The stagnation periods were chosen to allow sufficient time for contaminants to accumulate. Stagnant conditions represent the “worst case scenario” and the stagnation periods meet the EPA LCR requirement of “at least 6 h stagnation” for sampling at point of use. The longer the stagnation time, the more accumulation there is. The relative long stagnation period is likely to be encountered for residents returning from weekend short breaks or longer vacations. The sample in each bottle represents water was held stagnant in different segments (approximately 80 cm and 40 cm of pipe length for 100 mL and 50 mL, respectively) along the water pipe, starting from the tap. Only the first 200 mL was collected to minimize the dilution effect caused by the influx of fresh water during sampling, as there were multiple sampling locations (e.g., taps) in the system. The use of glass bottles instead of plastic bottles eliminated the need to transfer samples for acid digestion, solving the problem of underestimation of lead levels due to missed particulates which attached to the inner surface of the bottle [31]. Whole bottle digestion was performed to prevent any loss during transfer. Following the sample collection, the water in the plastic container was replaced with a fresh supply of tap water and the system was flushed with at least 3 L of the newly supplied water to ensure all the water in the system has been replaced. Orthophosphate was added to the solution in water container during changeover. Sample solution in each glass bottle was tested for pH, total copper, total lead and total zinc concentrations. The flow rates during sampling complied with allowable flow rate for constant flow regulations set by the local government whereby a basin tap should not exceed 6 L/min to conserve water usage [32]. The average flow rates during sampling of the taps were below 3 L/min.

### 2.2. Analytical Methods

Total lead, copper and zinc (soluble + particulate) were measured using inductively coupled plasma mass spectrometer optical emission spectrometry (ICP-OES) in accordance with Standard Method 3125-B [33]. Prior to the measurement, the solutions were acidified with 5% (v/v) nitric acid and digested at 85 °C for at least 2 h. The solution pH value was measured using a pH meter (Horiba F-51) equipped with an Ag/AgCl probe following the three-point calibration. Scanning electron microscopy (SEM) and energy dispersive X-ray spectroscopy (EDX) (Nova^TM^ NanoSEM 230) were used to investigate the morphology and chemical composition of the formed scales, respectively.

## 3. Results and Discussion

### 3.1. Conditioning Phase

**Figure 3:**
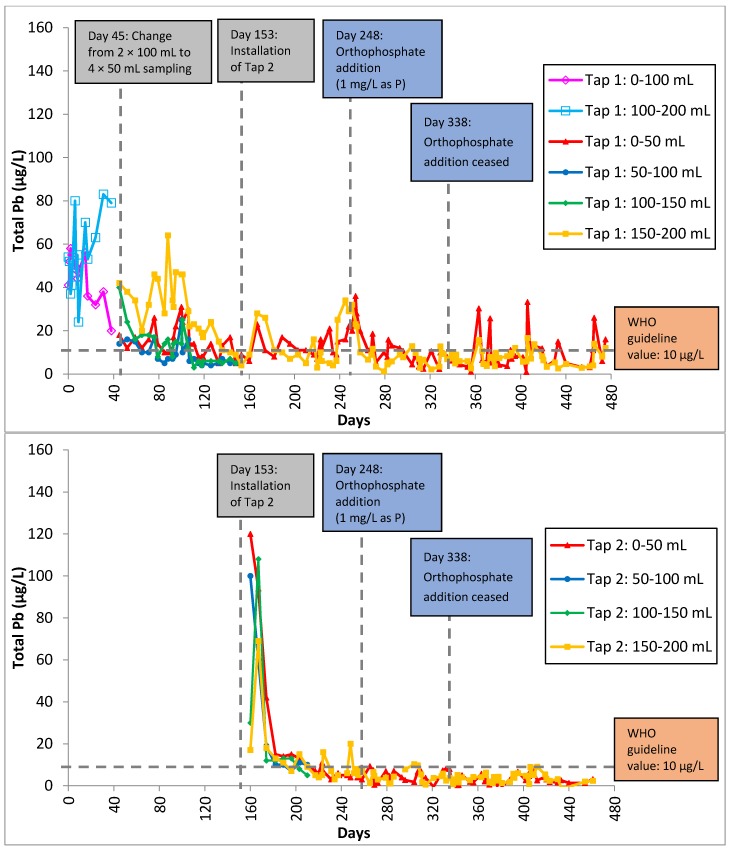
Total lead in Tap 1 (**top**) and Tap 2 (**bottom**) samples as a function of time for 475 days. A second tap was installed on Day 153 and orthophosphate was introduced on Day 248 and discontinued on Day 338.

Figure 3 shows the total lead release over the experimental period of 475 days. The system was first conditioned for 247 days, followed by orthophosphate addition for the next 89 days before the addition was ceased for the remaining 139 days. Total lead levels decreased gradually regardless of stagnation times (one, three and seven days), but exceeded the WHO guideline value of 10 µg/L for the first five months. The highest lead concentration recorded was 83 µg/L on Day 31. On Day 45, the sampling procedure for collecting the first 200 mL was changed from using 2 × 100 mL bottles to 4 × 50 mL bottles as the former were unable to accurately locate the lead source due to multiple brass fittings near the tap for accommodating the sharp bends and tight corners. A sampling volume of 100 mL is sufficient to detect lead contamination in drinking water in premise systems but an even smaller sampling volume may be used to provide a more accurate and precise location of the lead source. Brass fittings were identified to be the lead source in the system. The second and third 50 mL sequential samples were discontinued after 153 days because their lead concentrations were consistently lower compared to the other samples and they were below the guideline value of 10 µg/L, indicating absence of a lead source.

A second tap was installed on Day 153 and lead levels began to spike over the next two weeks. The scale on the inner surface of the pipes and brass fittings could have been destabilized during the installation, contributing to the lead spikes. The high lead levels may also be attributed to the dissolution of the surface lead of the brass fittings that were used to connect the copper pipes to the new tap. The high lead levels were not sustained for a long period of time, returning to pre-installation levels (~10 µg/L) after one month. The decrease of lead with time after the installation of the second tap may be due to either the removal of the destabilized scale during the tap installation or the surface passivation of the newly installed brass fitting, inhibiting further lead release into the solution. Surface lead may also have been gradually depleted, considering that most of the lead found in brass exists as surface contaminants [17]. Residents moving into new buildings or renovating their plumbing systems are potentially at risk of lead contamination in their drinking water. Lead contamination due to the presence of a lead source is expected to persist for as long as many years if the situation is left untreated or the system is left untouched. A study by Elfland [17] reported high lead levels in first flush samples in new buildings one and a half year after commissioning. Like many sampling studies, such studies can only show whether there is lead contamination at the point of sampling, but they are not able to explain if the lead contamination is an ongoing or short-term issue.

### 3.2. Orthophosphate Addition

The addition of orthophosphate is commonly used as a lead control measure to suppress lead levels in drinking water [34,35,36]. As expected, total lead decreased with the addition of orthophosphate (1 mg/L as P) from Day 248 to 337. The percentage of samples with total lead concentration less than 10 µg/L increased from 40% (85 out of 213 samples) without the orthophosphate addition to 77% (77 out of 100 samples) with the orthophosphate addition. While the orthophosphate addition can lead to the formation of relatively insoluble lead precipitates such as secondary lead phosphate (Pb_3_(PO_4_)_2_), hydroxypyromorphite (Pb_5_(PO_4_)_3_OH) and chloropyromorphite (Pb_5_(PO_4_)_3_)Cl) [37,38,39,40,41,42,43], it can also precipitate with soluble copper and zinc to form copper phosphate (Cu_3_(PO_4_)_2_, Cu_3_(PO_4_)_2_·2H_2_O) and zinc phosphate (Zn_3_(PO_4_)_2_, Zn_3_(PO_4_)_2_·4H_2_O), respectively [44,45]. The precipitation of insoluble phosphate minerals can control the soluble concentrations of various metals and inhibit further release by surface passivation. Occasional total lead spikes were observed throughout the duration and these spikes corresponded to samples collected after one day of stagnation. A closer look showed that the occurrences of the spikes shifted from samples further away from the tap to those nearer to the tap. After orthophosphate addition ceased, lead spikes occurred in Tap 1: 0–50 mL samples, while before orthophosphate was introduced, lead spikes occurred in Tap 1: 150–200 mL samples. This phenomenon could be attributed to the fast precipitation that was induced with the introduction of orthophosphate. Hence, it is likely that the precipitates may have been carried along the system, causing the segment nearer to the tap to contain more lead. The corrosion products accumulated at the tap over time can also be potential sources of lead, which could be destabilized during phase transformation when orthophosphate is introduced. Lead phosphate minerals, especially pyromorphite, are stable under *in vivo* conditions and produce the least bioavailable lead compared to other lead minerals such as lead carbonates [46]. Hence, using a phosphate-containing corrosion inhibitor can not only control soluble lead levels, but significantly reduce lead bioavailability. To minimize ingestion of lead-containing minerals, consumers should avoid flushing taps at maximum flow velocity (tap fully open) as fast-flowing water can promote physical corrosion within the premise distribution system and is more likely to carry the loose particles out of the taps. Unlike the taps used in a wash basin or wash cabinet, taps used for water consumption should come with an aerator to filter out any particles.

**Figure 4:**
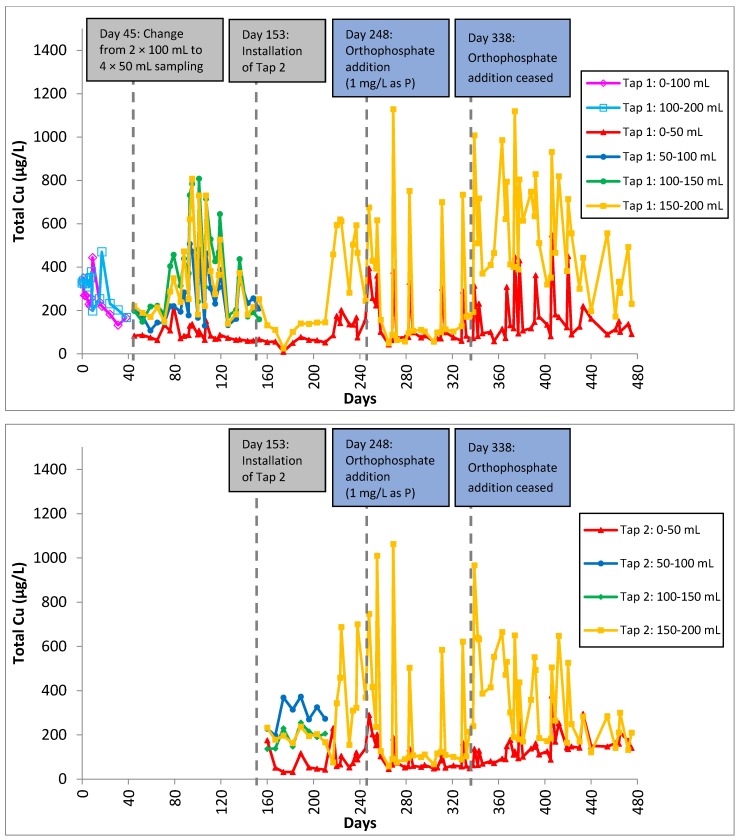
Total copper in Tap 1 (**top**) and Tap 2 (**bottom**) samples as a function of time for 475 days. A second tap was installed on Day 153 and orthophosphate was introduced on Day 248 and discontinued on Day 338.

**Figure 5:**
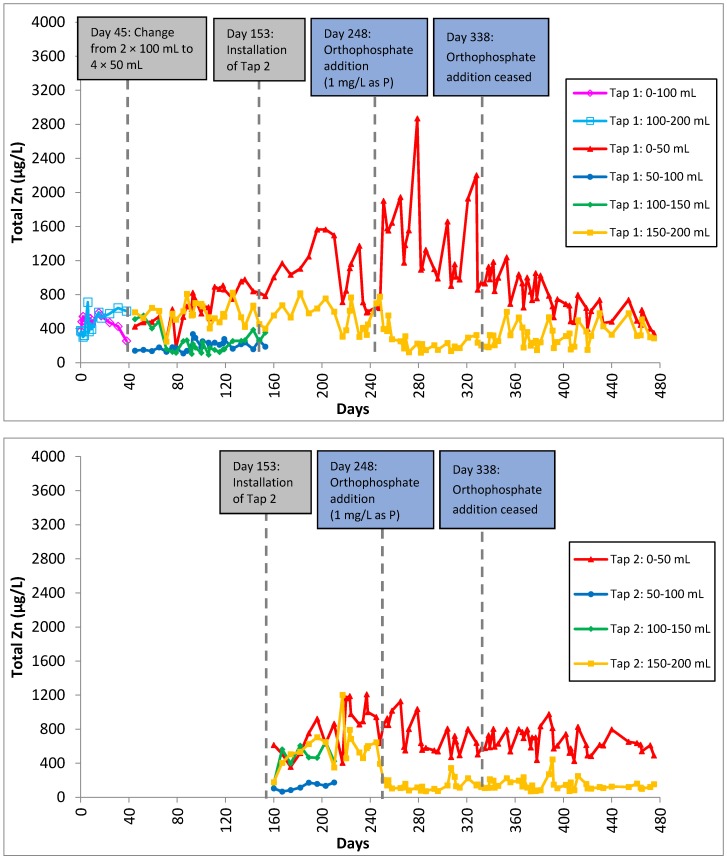
Total zinc in Tap 1 (**top**) and Tap 2 (**bottom**) samples as a function of time for 475 days. A second tap was installed on Day 153 and orthophosphate was introduced on Day 248 and discontinued on Day 338.

Total copper was significantly higher in segments further away from the tap (150–200 mL) than those near the tap (0–50 mL). This is due to the larger copper surface area in copper pipes and brass fittings in contact with water than at the tap. Total copper levels exhibited peaks during orthophosphate addition and these peaks also corresponded to samples with one day of stagnation (Figure 4). Copper-containing scale might become loose and detach from the surface when water is flushed through the system after the last sampling. Total copper decreased gradually after orthophosphate addition was ceased as the copper phosphate minerals were eventually flushed out. Two out of four segmental samplings showed that total zinc levels were suppressed with the addition of orthophosphate (Figure 5). 

Unlike copper, total zinc was significantly higher in segments (0–50 mL) near the tap when orthophosphate was introduced. Zinc could be potentially released from the taps as zinc was added to protect other metals such as copper, iron or steel against corrosion. Total zinc in one segment (Tap 1: 0–50 mL) increased with the introduction of orthophosphate while the other segments were unaffected, indicating that they were sources of dezincification, a form of galvanic corrosion where zinc is preferentially corroded over other metals such as copper and lead. The presence of orthophosphate accelerated dezincification, resulting in more zinc release. This finding is consistent with works by Lucey [47], Oliphant and Shock [48] and Oliphant [49]. After orthophosphate addition has ceased, total Zn returned to the levels before orthophosphate was introduced.

**Figure 6:**
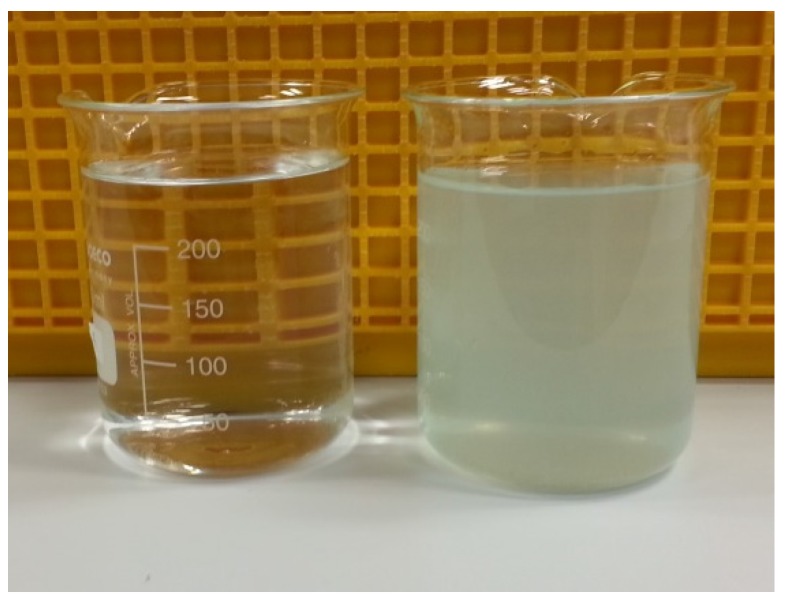
“Blue water” sample, before (**right**) and after filtration (**left**) with 0.45-micron pore-size nylon membrane.

“Blue water” (Figure 6) was also observed for samples with one day of stagnation. A clear, colorless filtrate was obtained when the “blue water” was filtered using a 0.45-micron pore-size nylon membrane, suggesting that the blue colorization was due to the predominant presence of copper minerals rather than soluble copper ions. The blue colorization most likely resulted from the presence of copper phosphate since “blue water” was only observed when orthophosphate was added. This finding supports the occurrence of peaks seen in total copper release during orthophosphate addition (Figure 4). Therefore, there should be caution when using orthophosphate for corrosion control if the premise plumbing system contains copper pipes. Further studies may be conducted to examine means to mitigate the formation of “blue water” with respect to the optimum dose of orthophosphate.

After orthophosphate addition had been ceased, total lead levels were seen to spike within a week (Figure 3). The presence of lead phosphate precipitate formed previously was not able to control lead release without a continuous supply of orthophosphate into the system. Total copper concentration increased gradually for all samples (Figure 4), suggesting that the formation of copper phosphate minerals was not effective in controlling copper levels as well. This observation is consistent with the study by Zhang and Edwards [50], who reported that the inhibitive effect of dosing orthophosphate decreased substantially after 100 days. However, total zinc continued to decrease even after orthophosphate addition had been stopped (Figure 5).

### 3.3. Change in Solution pH

The change in pH was obtained by taking the difference between the pH in the water sample collected after the desired stagnation time and the pH of the same water first introduced to the system. Changes in the pH value were shown to increase at each phase of the experiment (Table 2). During the conditioning phase, 82% of samples (112 out of 136 samples) collected showed an increase of at least 0.50 pH unit. The increase in pH could be caused by various chemical processes in the system. For instance, lead can be first oxidized to Pb^2+^ (Equations (1) and (2)), which subsequently reacts with oxygen to form an oxide layer. This basic oxide layer reacts with H^+^ ions to release soluble Pb^2+^ ions (Equation (3)). Both the oxidation of metallic Pb to Pb^2+^ and the dissolution of PbO to Pb^2+^ in water either consume H^+^ or produce OH^−^, resulting in an increase in solution pH.

(1) $$2\mathrm{Pb}+O_{2}+{4H}^{+}\to 2{\mathrm{Pb}}^{2+}+2H_{2}O$$

(2) $$2\mathrm{Pb}+O_{2}+{2H}_{2}O\to 2{\mathrm{Pb}}^{2+}+4{\mathrm{OH}}^{-}$$

(3) $$\mathrm{PbO}+{2H}^{+}\to {\mathrm{Pb}}^{2+}+H_{2}O$$

The extent of median pH increase rose further from 0.75 to 1.47 pH unit when orthophosphate was introduced into the system. However, this increase in median pH returned to the pre-orthophosphate level at 0.86 pH unit once the supply of orthophosphate was stopped. Ninety-six out of 100 samples collected had a pH increase between 1.21 and 1.92 pH unit when orthophosphate was added to the system while 92 out of 132 samples had a pH increase between 0.60 and 1.20 pH unit when the orthophosphate addition was stopped. The formation of phosphate minerals would deplete the soluble metal ions in the system and drive the system to produce additional soluble metal ions to replace the consumed ions. More H^+^ will be consumed in the process, resulting in a rise in pH. The different stagnation times had little effect on pH changes in each phase.

### 3.4. Scale Analysis

**Figure 7:**
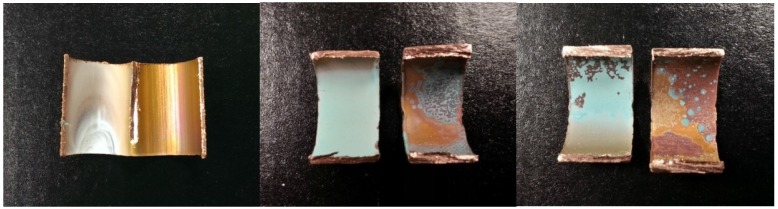
Cross-section of a copper pipe segment (**left**) after conditioning with tap water for 100 d, (**middle**) after 367 d orthophosphate addition and (**right**) after 433 d orthophosphate addition ceased.

**Figure 8:**
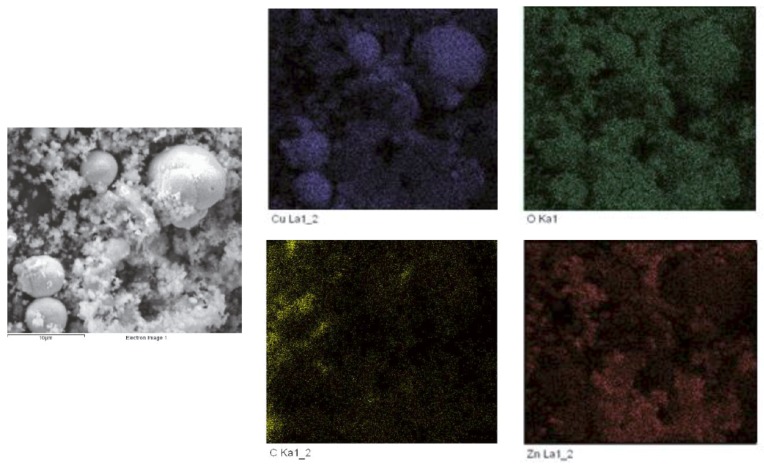
SEM-EDX elemental mapping of scale collected at the end of experiment.

Bluish-green scale (Figure 7) was observed on the copper pipe segments collected during orthophosphate addition and when orthophosphate addition was ceased. Copper corrosion products, such as copper hydroxide, carbonate and phosphate, were likely to be present, as evidenced by the same bluish-green coloration of these minerals and the scale. A cross-section of the segment showed the transformation in color of the scales from white to bluish-green when orthophosphate was introduced. Elemental mapping using SEM-EDX (Figure 8) identified the spherical crystals to be CuO and the smaller amorphous crystals to be ZnO.

## 4. Conclusions 

In this study, a modified sampling protocol was used to detect lead contamination in a premise plumbing system and locate lead sources within the system. The sampling protocol requires sequential sampling of 50 and 100 mL for the first 200 mL after a stagnation period of one, three or seven days. Sequential sampling using 100 mL was sufficient for detecting lead contamination while using 50 mL could effectively locate the lead source. “Lead-free” brass fittings were identified as the source of lead contamination. During the first 247 days of conditioning, lead levels far exceeded the WHO guideline value of 10 µg·L^−1^. Tap installation on Day 153 caused short-term spikes in lead release for two weeks. Hence, residents moving into new buildings or renovating their plumbing systems are potentially at risk of lead contamination in their drinking water. Orthophosphate was effective in suppressing total lead levels below 10 µg·L^−1^, but caused “blue water” problems, most likely due to excessive copper phosphate formation. When orthophosphate addition was ceased, total lead levels began to spike within one week, suggesting that a continuous supply of orthophosphate was required to control total lead levels. All samples collected after stagnation showed an increase in pH while orthophosphate addition resulted in the highest pH increase of up to 1.92 pH unit. The main strengths of this study are the measurement of copper and zinc on top of lead and the long experimental period. Copper and zinc are major components of brass, while lead is usually present in negligible amounts. The release of lead from brass is, however, related to zinc content in brass, *i.e*., the higher the zinc content, the higher the lead release [51]. The system was assembled in the laboratory using all-new plumbing materials and only certified copper pipes, brass fittings and stainless steel taps were used without any tapes, sealants and solders that may be present in field sampling. Many studies in the literature used either relatively short lengths of pipe (3 m) or single fittings to represent real distribution systems, while this study is specifically designed to represent real premise plumbing systems that use copper pipes. The long experimental period allows us to show that lead contamination can be persistent in premise distribution systems and helps to fill the knowledge gap in the existing literature. It should be noted that the findings presented in this study are specific to premise systems made up of primarily copper pipes and brass fittings and may differ from systems of different materials or dimension. The approach in tracing lead sources (or other contaminants) in a premise system by performing sequential sampling and adjusting sample volumes should still work for other systems. This study can be further improved by incorporating the measurement of soluble lead concentration to differentiate the contribution of soluble and particulate lead to the total lead level, adopting a higher sampling flow rate to investigate the potential high contribution of particulate lead to lead contamination, and using shorter stagnation periods to represent the daily usage of water.

## Figures and Tables

**Table 1:** Tap water parameters.

Water Parameter	Value
pH	7.7 ± 0.3
Total alkalinity	16 ± 0.9 mg/L as CaCO_3_
Chloride	35 ± 2.7 mg/L as Cl^−^
Sulfate	18 ± 2.0 mg/L as ${\mathrm{SO}}_{4}^{2-}$
Copper	17.2 ± 0.4 µg/L
CSMR (Chloride-to-sulfate mass ratio)	1.9
Lead	2.0 ± 0.6 µg/L
Free chlorine residual	<0.01 mg/L as Cl_2_
Monochloramine residual	0.02 ± 0.01 mg/L as Cl_2_
TOC	0.1 mg/L

**Table 2:** Change in pH values with respect to initial tap water pH in samples collected at different stages from the system.

Sequential Sample	Conditioning				Orthophosphate (1 mg/L as P)				Orthophosphate Addition Ceased			
	1 d	3 d	7 d	Median Min, Max	1 d	3 d	7 d	Median Min, Max	1 d	3 d	7 d	Median Min, Max
Tap 1: 0–50 mL	0.47 −0.09, 0.97	0.89 0.34, 1.60	0.73 0.36, 1.46	0.64 −0.09, 1.60	1.55 1.50, 1.79	1.59 1.27, 1.92	1.36 1.26, 1.57	1.55 1.26, 1.92	0.97 0.74, 1.83	1.01 0.48, 1.44	0.73 0.23, 0.97	0.97 0.23, 1.83
Tap 1: 50–100 mL	0.24 −0.21, 0.57	0.64 0.32, 0.80	1.11 0.83, 1.30	0.80 −0.21, 1.30								
Tap 1: 100–150 mL	0.16 −0.22, 0.57	0.65 0.38, 0.86	0.78 0.64, 0.87	0.68 −0.22, 0.87								
Tap 1: 150–200 mL	0.53 0, 0.97	0.83 0.50, 1.00	0.77 0.41, 1.16	0.75 0, 1.16	1.45 1.34, 1.72	1.44 0.89, 1.69	1.30 1.23, 1.50	1.42 0.89, 1.72	0.97 0.85, 1.64	0.85 0.41, 1.47	0.33 0.17, 0.78	0.83 0.17, 1.64
Tap 2: 0–50 mL	0.77 0.56, 0.97	0.56 0.36, 0.85	1.15 0.52, 1.73	0.97 0.36, 1.73	1.29 1.13, 1.50	1.48 1.29, 1.87	1.38 1.25, 1.72	1.44 1.13, 1.87	0.71 0.51, 1.56	0.84 0.46, 1.15	0.52 0.40, 0.92	0.76 0.40, 1.56
Tap 2: 50–100 mL			0.95 0.59, 1.14	0.95 0.59, 1.14								
Tap 2: 100–150 mL			0.75 0.65, 1.07	0.75 0.65, 1.07								
Tap 2: 150–200 mL	0.82 0.66, 0.97	0.85 0.70, 1.22	0.90 0.52, 1.24	0.89 0.52, 1.24	1.52 1.37, 1.60	1.46 0.87, 1.84	1.30 1.22, 1.50	1.42 0.87, 1.84	1.06 0.85, 1.67	0.96 0.56, 1.51	0.63 0.41, 0.94	0.90 0.41, 1.67
Median				0.75 −0.22, 1.73				1.47 0.87, 1.92				0.87 0.17, 1.83

## Abbreviations

The following abbreviations are used in this manuscript:

AS/NZS: Australian/New Zealand Standard
BS EN: British Standards in English language
CP: Code of Practice
CSMR: chloride-to-sulfate mass ratio
ICP-OES: Inductively coupled plasma mass spectrometer optical emission spectrometry
IRIS: Integrated Risk Information System
LCR: Lead and Copper Rule
LSLs: lead service lines
MCLG: Maximum Contaminant Level Goal
PUB: Public Utilities Board
RDT: random daytime
RfD: reference dose
USEPA: United States Environmental Protection Agency
WHO: World Health Organization
